# SRH and HrQOL: does social position impact differently on their link with health status?

**DOI:** 10.1186/1471-2458-12-19

**Published:** 2012-01-10

**Authors:** Cyrille Delpierre, Michelle Kelly-Irving, Mette Munch-Petersen, Valérie Lauwers-Cances, Geetanjali D Datta, Benoît Lepage, Thierry Lang

**Affiliations:** 1 Inserm, UMR1027, Toulouse, F-31073, France; 2 Department of public health science, Copenhagen University, Copenhagen, Denmark; 3 CHU Toulouse, Department of Epidemiology and Clinical Research, F-31073, France; 4 Department of Social and Preventive Medicine, Université of Montréal, Montreal, Canada; 5 Research Center of the Centre hospitalier de l'Université de Montréal, Montreal, Canada; 6 Université de Toulouse III, UMR1027, Toulouse, F-31073, France

**Keywords:** Subjective health indicators, Self-rated health, Quality of life, Socioeconomic position, Health inequalities

## Abstract

**Background:**

Self-rated Health (SRH) and health-related quality of life (HRQoL) are used to evaluate health disparities. Like all subjective measures of health, they are dependent on health expectations that are associated with socioeconomic characteristics. It is thus needed to analyse the influence played by socioeconomic position (SEP) on the relationship between these two indicators and health conditions if we aim to use them to study health disparities. Our objective is to assess the influence of SEP on the relationship between physical health status and subjective health status, measured by SRH and HRQoL using the SF-36 scale.

**Methods:**

We used data from the French National Health Survey. SEP was assessed by years of education and household annual income. Physical health status was measured by functional limitations and chronic low back pain.

**Results:**

Regardless of their health status, people with lower SEP were more likely than their more socially advantaged counterparts to report poor SRH and poorer HRQoL, using any of the indicators of SEP. The negative impact of chronic low back pain on SRH was relatively greater in people with a high SEP than in those with a low SEP. In contrast, chronic low back pain and functional limitations had less impact on physical and mental component scores of quality of life for socially advantaged men and women.

**Conclusions:**

Both SRH and HRQoL were lower among those reporting functional limitations or chronic low back pain. However, the change varied according SEP and the measure. In relative term, the negative impact of a given health condition seems to be greater on SRH and lower on HRQoL for people with higher SEP in comparison with people with low SEP. Using SRH could thus decrease socioeconomic differences. In contrast using HRQoL could increase these differences, suggesting being cautious when using these indicators for analyzing health disparities.

## Background

Self-rated health (SRH) [1] and health-related quality of life (HRQoL) [2], which is defined as the perception of the impact of health problems on different spheres of life, including physical, mental, and social aspects, are two outcome measures used to assess health status. Because they are self-reported, they are inexpensive and easy to use, and it has been shown that SRH [1,3,4] and in a lesser measure HRQoL [5-9], are independent predictors of subsequent mortality and morbidity. As a consequence, SRH and more recently HRQoL [10,11], have been used as an alternative to mortality or morbidity for measuring health disparities. As an example, Mackenbach et al. compared social inequalities in health between 22 European countries using mortality and SRH as health measures [12]. Interestingly, the magnitude of social inequalities was greater by using mortality rather than SRH. By considering mortality as the "gold standard" for measuring social inequalities in health, these results suggest that using SRH as measure of health might underestimate social disparities in health.

Contrary to mortality, SRH and HRQoL are both dependent on individual expectations. SRH can be considered as a balance between one's actual health and the best health that one could expect for oneself [13], while HRQoL refers to the physical, psychological and social domains of health which are influenced by a person's experiences, beliefs, expectations and perceptions [2]. The way people rate their health depends therefore on their expectations of what their health should be. The expectations that people have vary according to several factors, such as socioeconomic position (SEP) and cultural or social issues [14,15], which may bring about differences in reporting health status or quality of life for the same health condition. As expectations seem to be higher among people with high SEP [16], the same disease may have a more negative impact on SRH among them than among people with low SEP. In fact, recent studies conducted in the U.S. have shown that the relative impact on SRH of a given chronic condition was greater in people with a higher years of education than in those with less education [17-19], suggesting that using SRH as a measure of health could lead to underestimation of the magnitude of health inequalities existing between socioeconomic groups, as observed by Mackenbach et al. [12]. At the opposite, some others studies conducted on Canadian samples have shown no such interaction or a relative lower impact of health condition on SRH [20,21].

As suggested by Smith et al. "understanding if people from different SES background interpret levels of SRH differently is essential for..... comparing health inequalities with SRH" [21]. This type of variation by SEP may be relevant for subjective health indicators other than SRH as well. One such indicator is the SF-36 scale which is a general questionnaire measuring health-related quality of life through its physical and mental components. It has been shown that SRH was associated with all dimensions of the SF-36 [22], suggesting that SRH may include perceptions of a range of physical, mental and social factors. Because SRH and the SF-36 scale measure related concepts and because SRH seems to vary according to SEP given health status, exploring the potential influence of SEP on HRQoL may be of interest.

Data on the influence of SEP on the relationship between HRQoL and health conditions are limited. However Sacker et al. have shown that the negative impact of coronary heart disease on SF-36 was greater among lower-grade civil servants, suggesting that, contrary to what is observed with SRH, the negative impact of a given health condition may be higher for people in a low socioeconomic position [23]. The influence of SEP on the relationship between health conditions and subjective health may be thus different according to indicators used to measure subjective health. The aim of this study was thus to assess if the SEP had an influence on the relationship between health conditions and subjective health status, measured by SRH and an indicator of HRQoL, the SF-36 questionnaire, and to assess if this influence was different on SRH and on SF-36.

## Methods

### Study population and sample design

The French data comes from the National Health Survey (NHS) (http://www.cnis.fr/ind_doc.htm), the only source of systematic statistical data on the health, health-care consumption and socioeconomic characteristics of French households. This study has been described in detail elsewhere [24]. Briefly, data were collected through a multilevel, stratified, random survey of households that, on the basis of data from the 1999 national population census, are representative of the French population. People who live in institutions (e.g. retirement homes, religious communities, prisons and hospitals), in mobile homes or who are homeless are not included, therefore almost 98% of the entire population is covered by the survey [24]. Using a combination of face-to-face interviews and self-administered questionnaires, the NHS includes data at both individual and household levels, including information about demographic and socioeconomic characteristics and health status, complementary insurance coverage and medical care consumption.

A new data collection was carried out between October 2002 and September 2003 and constitutes our sample. People were interviewed in five waves, throughout the year to account for seasonal variability. Three face-to-face interviews were conducted at one-month intervals. A self-administered health questionnaire was given to each participant after the first visit and was collected at the second or the third visit. The overall response rate during the 2003 NHS survey was > 85%. The global sample was composed of 16,821 households, representing 40,796 individuals.

We excluded participants younger than 18 years old before conducting analyses (N = 9800). Among the 30996 adults, we restricted our analyses to participants who responded to the three visits and who completed the self-administered health questionnaire (N = 26341). We then excluded those who were considered by the interviewer as not able to complete the questionnaire (n = 513). We also excluded people claiming full state health-care coverage (pregnant women, people with serious and high-cost disease, disabled persons) who may represent a sub population with specific health expectations compared with general population due to the existence of severe disease (n = 4043). The final sample consisted of 10,093 men and 11,692 women.

### Socioeconomic position

SEP was assessed by using two indicators: years of education, categorized as less than 12 years, 12 years, and more than 12 years and annual household income per consumption unit. Annual household income per consumption unit corresponds to the total income reported within the household divided by the number of consumption units of the household. The OCDE scale gives a weight of 1 to the first member of the household, a weight of 0.5 for any other adult and a weight of 0.3 for any child of less than 14 years. It was categorized in 4 classes according to quartiles (< Euro 9,900; 9,900-14,300, 14,300-20,400, > = Euro 20,400)

### Physical health conditions

We considered physical health conditions available in the study data which are known to be strong determinants of SRH and quality of life: functional limitations and chronic low back pain [22,25,26]. These two health conditions were not measured in an "objective" way but used diagnostic validated questionnaire.

Functional limitations (FL) were self-reported and assessed with the activities of daily living scale (ADLs), instrumental activities of daily living scale (IADLs), mobility and upper/lower body strength [27]. Participants who reported some difficulty, much difficulty or who were unable to do one of the activities were considered to have FL.

Chronic low back pain was assessed through a validated self-administered questionnaire, the French version of the Nordic questionnaire for the analysis of musculoskeletal symptoms [28]. This questionnaire includes 4 questions on presence and duration of low back pain during the past year before study, and type of pain. Participants who reported at least one event of low back pain for more than 30 days (at least 30 days but not daily, or pain everyday) were considered as having chronic low back pain [29].

### Subjective health status

Subjective health status was evaluated with two indicators: Self-rated health and health-related quality of life.

SRH ("How is your general health?") was measured by using the WHO recommended version asking participants to rate their health as very good, good, fair, poor or very poor. The responses were dichotomized in our analyses: individuals reporting very good or good health were classified as having good SRH and those reporting fair or poor or very poor health as having poor SRH.

HRQoL was measured with the SF-36 scale. The SF-36 has been validated and described in detail elsewhere [30]. It covers issues relating to physical, psychological and social functioning and is coded into eight scales: general health perceptions (5 items), physical functioning (10 items), role limitations due to physical functioning (4 items), bodily pain (2 items), general mental health (5 items), role limitations due to emotional problems (3 items), vitality (4 items) and social functioning (2 items). The remaining item relating to change in health is not scored as a separate dimension. These eight scales can be summarized into physical and mental functioning component scores (the Physical Component Summary (PCS), and the Mental Component Summary (MCS)) [30], which were used as indicators of quality of life in the study. Jenkinson suggested US scoring be adopted throughout the world [31]. Thus, the PCS and MCS were scaled using general US population norms to have mean +/- standard deviation values of 50 +/- 10. A higher score indicates a better quality of life.

### Statistical analysis

SEP may operate differentially on subjective health in men and women [32-36], and according to age. Therefore, all our analyses were run separately for men and women and all were adjusted on age.

We were interested in studying whether education or income could modify the association between SRH/HRQoL and health conditions, and thus influence the measure of health inequalities by using subjective measures of health. Therefore we focused on the interaction effect between socioeconomic position and health conditions on SRH and HRQoL.

Regarding SRH, to test this interaction, we constructed logistic regression models with the probability of reporting poor SRH as the outcome and included terms for socioeconomic position (education and income separately), the health condition, and the interaction between education and the health condition (example of the model with education and FL: poor SRH = Age + FL + education + education*FL). As a statistical interaction was detected for most conditions, the results were presented after stratification on socioeconomic position (education and income). In tables, the relationship between health conditions and SRH was analyzed and presented using logistic regression adjusted on age, for each socioeconomic group.

Using the same methodological approach, with MCS and PCS as the outcome, we constructed multiple linear regression models that included terms for each SEP indicator separately, the health condition, and the interaction between the SEP indicator and the health condition. Again as a statistical interaction was detected for most conditions, the results were presented after stratification on socioeconomic position. In tables, the relationship between health conditions and PCS/MCS was analyzed and presented using multiple linear regression models adjusted on age, for each socioeconomic group.

We used sampling weights to produce our weighted estimates and sampling errors (SEs). Sampling weights were used to correct for systematic nonresponse bias. This procedure allows data to be weighted in an inversely proportional relationship to the nonresponse probabilities of individuals to the survey and the self-administered questionnaires, in the aim to perfectly reflect the French population.

Statistical analyses were performed using SAS (version 9.1, SAS Institute, Cary, NC).

## Results

### Socioeconomic position and health

The social gradient was associated with poorer SRH and poorer quality of life in both men and women (Table 1). Men and women with lower years of education were more likely to have FL and chronic low back pain compared with those with more than 12 years of education (Table 1). Men with a lower level of income were more likely to have FL and chronic low back pain than those with higher level of income. Women with a lower level of income were more likely to have FL than those with higher level of income, but no gradient was observed for chronic low back pain. Social gradient was steeper for FL than for chronic low back pain, with both socioeconomic indicators (education and income).

**Table 1:** Relationships between Socioeconomic Position and Health, in Men and Women; National Health Survey

	Poor SRH (%)	PCS (Mean)	MCS (Mean)	Functional limitations (%)	Chronic low back pain (%)
	
	MEN				
Education (missing = 720)					
	
< 12 years (n = 2432)	26.1	50.2	50.3	14.4	15.3
12 years (n = 3688)	17.9	51.5	50.4	10.1	15.7

> 12 years (n = 3253)	10.6	53.3	51.3	7.6	12.3

P-value*	< 0.0001	< 0.0001	0.009	< 0.0001	0.001

Missing (N)	6	1747	1747	3	1611

Annual income (€)					

< 9,900 (n = 2380)	24.8	50.5	49.7	14.1	15.2

9,900-14,300 (n = 2515)	19.6	51.7	50.3	10.9	13.8

14,300-20,400 (n = 2529)	14.4	52.3	50.9	8.0	14.2

> = 20,400 (n = 2669)	8.4	53.6	51.3	6.4	11.8

P-value*	< 0.0001	< 0.0001	< 0.0001	< 0.0001	0.02

Missing (N)	7	1855	1855	3	1723

	**WOMEN**				
	
Education (missing = 784)					

< 12 years (n = 3650)	30.5	48.8	47.7	24.7	19.6

12 years (n = 3768)	22.1	51.2	47.7	16.5	20.1

> 12 years (n = 3490)	16.4	52.0	48.4	14.5	16.2

P-value*	< 0.0001	< 0.0001	0.02	< 0.0001	0.001

Missing (N)	5	2131	2131	2	1924

Annual income (€)					

< 9,900 (n = 3092)	28.1	49.7	46.8	22.8	17.7

9,900-14,300 (n = 2981)	23.4	50.5	47.4	19.4	19.3

14,300-20,400 (n = 2830)	19.7	51.7	48.4	15.9	18.2

> = 20,400 (n = 2789)	15.4	52.1	48.8	10.6	17.4

P-value*	< 0.0001	< 0.0001	< 0.0001	< 0.0001	0.39

Missing (N)	5	2211	2211	3	2016

SRH: Self Rated Health; PCS: SF-36 Physical Component Summary; MCS: SF-36 Mental Component Summary % adjusted for age

*Global chi-square

### Interaction of socioeconomic position and health conditions on SRH (Tables 2 and 3)

**Table 2:** Proportion of People Reporting Poor SRH According to the Presence or Absence of Health Conditions by Socioeconomic Position, Among Men; National Health Survey

	Years of education				
	
	< 12 years	12 years		> 12 years	Interaction test
	
Functional limitations, %^a^					
No	20.7	14.1		8.1	0.33^b^

Yes	58.5	52.8		36.7	0.40^c^

OR (95% CI)^†^	4.5 (3.3-6.1)	5.5 (4.0-7.5)		5.2 (3.4-7.9)	

					

Chronic low back pain, %^a^					

No	22.6	13.8		8.0	0.12^b^

Yes	47.8	40.0		28.0	0.05^c^

OR (95% CI)^†^	2.9 (2.1-4.0)	4.0 (3.1-5.2)		4.6 (3.2-6.6)	

	**Income level (€)**				
	
	< 9,900	9,900-14,300	14,300-20,400	> = 20,400	Interaction test
	
Functional limitations, %^a^					

No	18.4	15.2	12.1	6.3	0.42^d^

Yes	66.0	56.4	40.4	38.4	0.03^e^

					0.77^f^

OR (95% CI)^†^	6.1 (4.1-9.0)	4.8 (3.3-6.8)	3.1 (2.1-4.8)	6.2 (4.0-9.5)	

Chronic low back pain, %^a^					

No	19.7	16.2	11.2	6.0	0.09^d^

Yes	55.3	39.5	33.3	25.7	0.24^e^

					0.59^f^

OR (95% CI)^†^	4.7 (3.2-6.7)	3.0 (2.1-4.3)	3.5 (2.5-4.9)	4.1 (2.8-5.9)	

^a^: % adjusted for age

^b^: Interaction between health status and years of education: results for years of education = 12 years compared with years of education < 12 years; model adjusted for age

^c^: Interaction between health status and years of education: results for years of education > 12 years compared with years of education < 12 years; model adjusted for age

^d^: Interaction between health status and income level: results for income level between 9,900-14,300 **€ **compared with income level < 9,900 **€**; model adjusted for age

^e^: Interaction between health status and income level. Results for income level between 14,300-20,400 **€ **compared with income level < 9,900 **€**; model adjusted for age

^f^: Interaction between health status and income level. Results for income level > = 20,400 **€ **compared with income level < 9,900 **€**; model adjusted for age.

† Odds ratio (95% confidence interval) from logistic regression models including health conditions and age in each socioeconomic group; reference group = no health condition

**Table 3:** Proportion of People Reporting Poor SRH According to the Presence or Absence of Health Conditions by Socioeconomic Position, Among Women; National Health Survey

	Years of education				
	
	< 12 years	12 years		> 12 years	Interaction test
	
Functional limitations, %^a^					
No	21.3	15.8		11.3	0.18^b^

Yes	59.4	54.4		44.2	0.22^c^

OR (95% CI)^†^	4.0 (3.2-5.0)	5.9 (4.5-7.7)		5.3 (3.8-7.3)	

					

Chronic low back pain, %^a^					

No	26.1	17.1		13.6	0.12^b^

Yes	47.4	42.3		28.5	0.75^c^

OR (95% CI)^†^	2.6 (2.0-3.3)	3.4 (2.7-4.2)		2.4 (1.8-3.2)	

	**Income level (€)**				
	
	< 9,900	9,900-14,300	14,300-20,400	> = 20,400	Interaction test
	
Functional limitations, %^a^					

No	18.4	15.8	13.8	11.6	0.52^d^

Yes	63.2	56.0	51.3	46.5	0.42^e^

					0.30^f^

OR (95% CI)^†^	5.5 (4.0-7.5)	4.5 (3.3-6.0)	4.6 (3.4-6.2)	4.1 (2.9-5.8)	

Chronic low back pain, %^a^					

No	24.3	19.6	14.4	11.0	0.93^d^

Yes	45.0	39.1	40.9	32.1	0.03^e^

					0.17^f^

OR (95% CI)^†^	2.4 (1.8-3.2)	2.4 (1.9-3.2)	3.8 (2.8-5.0)	3.2 (2.4-4.3)	

^a^: % adjusted for age

^b^: Interaction between health status and years of education: results for years of education = 12 years compared with years of education < 12 years; model adjusted for age

^c^: Interaction between health status and years of education: results for years of education > 12 years compared with years of education < 12 years; model adjusted for age

^d^: Interaction between health status and income level: results for income level between 9,900-14,300 **€ **compared with income level < 9,900 **€**; model adjusted for age

^e^: Interaction between health status and income level. Results for income level between 14,300-20,400 **€ **compared with income level < 9,900 **€**; model adjusted for age

^f^: Interaction between health status and income level. Results for income level > = 20,400 **€ **compared with income level < 9,900 **€**; model adjusted for age.

† Odds ratio (95% confidence interval) from logistic regression models including health conditions and age in each socioeconomic group; reference group = no health condition

FL and chronic low back pain were associated with poorer SRH using each indicator of SEP in both men and women.

Among men, regarding the influence of years of education, the relative impact of chronic low back pain on SRH was higher for those with more than 12 years of education than for those with less than 12 years (interaction test P = 0.05), as for those with 12 years of education, the interaction test being non significant (interaction test P = 0.12) (Table 2). In age-adjusted models, the relative increase in the proportion of men reporting poor SRH in the case of chronic low back pain was higher in the more highly educated group than in the lower educated group (respectively OR = 4.6, 95% CI 3.2 - 6.6 in men with more than 12 years of education and OR = 4.0, 95% CI 3.1 - 5.2 in those with 12 years of education vs OR = 2.9, 95% CI 2.1 - 4.0 in the least educated men).

Regarding the influence of income, the proportion of men reported poor SRH in case of FL increased relatively less in men with income between 14,300-20,400 € compared with the poorest men (OR = 3.1, 95% CI 2.1 - 4.8 in men with income between 14,300-20,400 € vs OR = 6.1, 95% CI 4.1 - 9.0 in men with income lower than 9,900 €, interaction test P = 0.03). No influence was observed for chronic low back pain.

Among women, regarding the influence of education, in age-adjusted model, the relative increase in the proportion of women reporting poor SRH in case of chronic low back pain was higher in those with 12 years of education than those with less than 12 years of education (OR = 3.4, 95% CI 2.7 - 4.2 for those with 12 years of education vs OR = 2.6, 95% CI 2.0 - 3.3 for the least educated), although the interaction test was not significant (P = 0.12) (Table 3). The relative increase of reporting poor SRH in case of FL were also higher for women with more than 12 years of education compared with the least educated women, interaction tests being not significant.

Regarding the influence of income, in age-adjusted models, the relative increase of reporting poor SRH in the case of chronic low back pain was higher in women with income between 14,300-20,400 € than in the poorest women (OR = 3.8, 95% CI 2.8 - 5.0 in women with income between 14,300-20,400 € vs OR = 2.4, 95% CI 1.8 - 3.2 in women with less than 9,900 €, interaction test P = 0.03).

### Interaction of socioeconomic position and health conditions on quality of life (Tables 4, 5)

Generally, for both men and women, PCS and MCS scores were lower when a health condition was present, whatever the SEP category.

Among men, regarding the influence of SEP on the relationship between health conditions and PCS score (Table 4), the decrease in PCS score associated with chronic low back pain was less pronounced for men with more than 12 years of education compared with those with less than 12 years of education (Interaction test P = 0.0002). The decrease of PCS score in the case of FL or chronic low back pain was also smaller in men with higher than 9,900 € compared with men with income lower than 9,900 €.

**Table 4:** HRQoL Score According to the Presence or Absence of Health Conditions by Socioeconomic Position, Among Men; National Health Survey

	PCS				
	**Years of education**				
	
	**< 12 years**	**12 years**		**> 12 years**	**Interaction test**
	
Functional limitations, mean^a^					

No	51.5	52.5		53.9	0.11^b^

Yes	42.4	42.4		45.7	0.31^c^

Regression coefficient^†^	-8.7	-10.3		-8.4	

					

Chronic low back pain, mean^a^					

No	51.5	52.7		54	0.28^b^

Yes	43.8	45.6		48.8	0.0002^c^

Parameter^†^	-7.6	-7.1		-5.4	

Interaction test					

	**Income level (€)**				
	
	< 9,900	9,900-14,300	14,300-20,400	> = 20,400	Interaction test
	
Functional limitations, mean^a^					

No	52.0	52.7	53.0	54.1	0.02^d^

Yes	41.2	43.6	45.1	45.3	0.0002^e^

					0.01^f^

Regression coefficient^†^	-10.8	-8.9	-7.9	-8.9	

Chronic low back pain, mean^a^					

No	51.8	52.6	53.2	54.4	0.004^d^

Yes	43.5	46.4	47.6	47.8	0.0002^e^

					0.02^f^

Regression coefficient^†^	-8.1	-6.1	-5.8	-6.6	

	**MCS**				

	**Years of education**				
	
	< 12 years	12 years		> 12 years	Interaction test
	
Functional limitations, mean^a^					

No	50.8	50.8		51.5	0.85^b^

Yes	47.2	47.0		49.3	0.12^c^

Regression coefficient^†^	-4.1	-3.5		-2.0	

Chronic low back pain, mean^a^					

No	50.8	50.9		51.8	0.85^b^

Yes	47.7	47.7		47.7	0.19^c^

Regression coefficient^†^	-3.1	-3.1		-4.2	

	**Income level (€)**				
	
	< 9,900	9,900-14,300	14,300-20,400	> = 20,400	Interaction test
	
Functional limitations, mean^a^					

No	50.4	50.6	51.2	51.4	0.11^d^

Yes	45.9	47.5	48.4	49.2	0.06^e^

					0.02^f^

Regression coefficient^†^	-4.6	-3.2	-2.8	-2.0	

Chronic low back pain, mean^a^					

No	50.3	50.8	51.4	51.7	0.31^d^

Yes	46.2	47.5	48.5	48.3	0.16^e^

					0.35^f^

Regression coefficient^†^	-4.0	-3.3	-3.1	-3.3	

^a^: mean adjusted for age

^b^: Interaction between health status and years of education: results for years of education = 12 years compared with years of education < 12 years; model adjusted for age

^c^: Interaction between health status and years of education: results for years of education > 12 years compared with years of education < 12 years; model adjusted for age

^d^: Interaction between health status and income level: results for income level between 9,900-14,300 **€ **compared with income level < 9,900 **€**; model adjusted for age

^e^: Interaction between health status and income level. Results for income level between 14,300-20,400 **€ **compared with income level < 9,900 **€**; model adjusted for age

^f^: Interaction between health status and income level. Results for income level > = 20,400 **€ **compared with income level < 9,900 **€**; model adjusted for age

† Results from multiple linear regression models adjusted on age; reference group = no health condition

The influence of SEP on the relationship between health conditions and MCS score was significant for men for whom the impact of FL on MCS score was lower in those earning more than 14,300 € compared with in men with the lowest income (Table 4). The same was observed in the case of FL for the most educated men compared with the east educated, but the interaction test was near to the significance threshold (*P *= 0.12).

Among women, regarding the influence of SEP on the relationship between health conditions and PCS score (Table 5), reporting chronic low back pain lowered the PCS score less among those with the highest years of education than in those with the lowest length (Interaction test *P *= 0.07). A similar trend was not observed with level of income.

**Table 5:** HRQoL Score According to the Presence or Absence of Health Conditions by Socioeconomic Position, Among Women; National Health Survey

	PCS				
	**Years of education**				
	
	**< 12 years**	**12 years**		**> 12 years**	Interaction test
	
Functional limitations, mean^a^					

No	51.2	52.8		53.3	0.36^b^

Yes	41.4	43.4		44.2	0.25^c^

Regression coefficient^†^	-9.4	-9.8		-9.2	

					

Chronic low back pain, mean^a^					

No	50.1	52.5		52.9	0.98^b^

Yes	43.8	46.2		47.7	0.07^c^

Parameter^†^	-6.1	-6.4		-5.4	

Interaction test					

	**Income level (€)**				
	
	< 9,900	9,900-14,300	14,300-20,400	> = 20,400	Interaction test
	
Functional limitations, mean^a^					

No	51.9	52.6	53.2	53.2	0.06^d^

Yes	42.3	42.1	44.0	43.4	0.51^e^

					0.69^f^

Regression coefficient^†^	-9.7	-10.3	-9.4	-9.6	

Chronic low back pain, mean^a^					

No	50.9	51.8	52.8	53.4	0.71^d^

Yes	44.7	45.8	47.2	47.0	0.33^e^

					0.76^f^

Regression coefficient^†^	-6.2	-5.9	-5.6	-6.4	

	**MCS**				

	**Years of education**				
	
	< 12 years	12 years		> 12 years	Interaction test
	
Functional limitations, mean^a^					

No	48.5	48.2		48.9	0.39^b^

Yes	45.0	45.3		45.0	0.57^c^

Regression coefficient^†^	-3.3	-3.0		-4.3	

					

Chronic low back pain, mean^a^					

No	48.2	48.6		48.8	0.37^b^

Yes	44.6	44.4		46.1	0.26^c^

Regression coefficient^†^	-3.5	-4.2		-2.9	

	**Income level (€)**				
	
	< 9,900	9,900-14,300	14,300-20,400	> = 20,400	Interaction test
	
Functional limitations, mean^a^					

No	47.8	48.0	48.8	49.0	0.07^d^

Yes	43.5	44.9	45.9	46.5	0.06^e^

					0.02^f^

Regression coefficient^†^	-4.3	-2.9	-2.9	-2.7	

Chronic low back pain, mean^a^					

No	47.7	47.9	49.0	49.4	0.02^d^

Yes	42.7	44.7	46.0	45.9	0.01^e^

					0.06^f^

Regression coefficient^†^	-4.9	-3.2	-3.0	-3.6	

^a^: mean adjusted for age

^b^: Interaction between health status and years of education: results for years of education = 12 years compared with years of education < 12 years; model adjusted for age

^c^: Interaction between health status and years of education: results for years of education > 12 years compared with years of education < 12 years; model adjusted for age

^d^: Interaction between health status and income level: results for income level between 9,900-14,300 **€ **compared with income level < 9,900 **€**; model adjusted for age

^e^: Interaction between health status and income level. Results for income level between 14,300-20,400 **€ **compared with income level < 9,900 **€**; model adjusted for age

^f^: Interaction between health status and income level. Results for income level > 20,400 **€ **compared with income level < 9,900 **€**; model adjusted for age.

† Results from multiple linear regression models adjusted on age; reference group = no health condition

Regarding the influence of SEP on the relationship between health conditions and MCS score, the decrease of MCS score in the case of chronic low back pain or FL was smaller in women with income higher than 9,900 € compared with women with an income below 9,900 €. No influence of education was observed.

## Discussion

To our knowledge, this is one of the first studies analyzing the influence of SEP on the relationship between physical health conditions and SRH on the one hand and a generic measure of HRQoL, the SF-36 questionnaire, on the other hand. Our results suggest that SEP influences the impact of health conditions, like FL or chronic low back pain, on subjective health in a different way according to whether it is measured by SRH or HRQoL. Compared with people with low SEP, some health conditions like chronic low back pain seem to have a greater negative impact on SRH in socially advantaged people, but the opposite occurred for quality of life. The strength of this interaction varied according to the indicator used to measure health conditions as well as the indicator used to define SEP.

An important limitation of our study is that health conditions were self-reported, and may be susceptible to misreporting [37]. However, we mainly studied chronic disabling diseases, assessed using valid questionnaires or a standardized definition that may be less susceptible to this type of misreporting [38]. Moreover, self reports could be reasonably accurate for certain chronic conditions [39,40]. Haapanen et al. showed that agreement between questionnaire data and medical records may be good for chronic diseases that have a clear definition [41]. As we used chronic health conditions and valid questionnaires or definitions to measure them, we believe that the proportion of misreporting is low, and unlikely to explain the opposite directions for SRH and HRQoL. Idler et al. showed that knowledge of a chronic illness strengthened the association between SRH and mortality [42]. Thus, use of patient-reported health conditions could constitute an appropriate indicator for analyzing the association between health conditions and subjective health. However, future studies are needed to examine the influence of SEP on the relationship between subjective health and objective health or "true health", assessed through more objective measures or by using multiple indicators linear structural equation models with latent variables as done by Shmueli et al. [43].

Another limitation is that some items used to evaluate FL are components of PCS score of the SF36 questionnaire. As low educated people have higher number of FL than high educated men, their PCS score should be poorer and could explain why PCS scores are poorer among lower educated people in case of disease. Although FL is subsumed within the concept of HRQoL measured by the SF-36 questionnaire, these two measures are not exactly the same. As an illustration, some works have shown that FL was a predictive factor of HRQoL, justifiying that FL and HRQoL are two different concepts [22,25,26]. In our study correlations between PCS score and FL were -0.38 in men and -0.49 in women. Therefore we do not think that this correlation is likely to explain totally the lower decrease of PCS score observed among people with high SEP. Moreover the same interaction is observed with MCS score for which no correlations were found between FL and MCS. Finally, we observed a lower decrease of PCS score for men with higher SEP in case of chronic low back pain, which is a different measure than the SF-36 questionnaire.

Another limitation is that tests of interaction have usually classically low power [44,45]. It is thus likely that some of interactions tests we performed lacked power to put in evidence a significant influence of SEP on the relationship between health status and subjective health.

In our study we used the SF-36 scale as a measure of HRQoL. As observed by Luo et al. health status assessed by different HRQoL indicators is not exactly the same [46]. Even more disturbing for the analysis of social inequalities in health, socioeconomic disparities may vary according to HRQoL indicators used to measure health and indicators used to measure socioeconomic position [11]. Therefore, studies exploring HRQoL with indicators other than the SF-36 are needed.

This study's main strength lies in the fact that the NHS is a national and representative sample and enabled measurement of socioeconomic position by using education and income.

Socially advantaged people were generally at less risk of having or reporting health problems. As expected, SRH and HRQoL were positively associated with SEP. Regardless of health conditions people with lower educational attainment or lower income were more likely than others to report poor SRH and to have poorer quality of life. This gradient was consistent using any of the indicators of SEP, in contrast to the observations of Robert et al. who found that income was more consistently associated with HRQoL and SRH measures among US adults [11].

The presence of a given health condition lowered reported levels of SRH and HRQoL, but the relative impact this condition had on SRH and on HRQoL was different. Regarding SRH, the influence of SEP on the relationship between chronic health conditions and SRH was not consistently significant, but this influence was mainly in the same direction: the impact of chronic health condition, like chronic low back pain, was relatively greater for socially advantaged people. Put differently, people with a high SEP were more likely to report a negative impact of this health condition on their SRH than those with a low SEP. One possible explanation of this finding is that a person's expectations about their health increase with increasing SEP [47]. The repercussions of health problems on SRH would therefore be worse for those with higher health expectations. Another possibility is that one's ability to be aware of one's own health status and to estimate risk is higher in socially advantaged people [48,49]. In the event of disease, they are more likely to be aware of the consequences of a health problem, in terms of morbidity or mortality risks, and thus more likely to report poor self-rated health.

In contrast, regarding quality of life, the impact of health conditions on PCS and MCS was lower for socially advantaged people. Shmueli et al. had also showed that, for a same "true health state" (true health considered as a latent variable), individuals in better economic status reported higher health related quality of life than individuals in poor economic status [50]. In our study, among men, the higher their income, the lower its impact on the PCS score, this interaction being less consistent with education. Among women, the same phenomenon was observed for education in case of chronic low back pain. It is noteworthy that income seems to have more influence on the relationship between health conditions and PCS score in men than in women. For MCS score, FL lessened the MCS score for the most highly educated and richest men. Among women, no influence of education was found but the impact on MCS score of chronic low back pain and FL was less pronounced in women with higher income. It is likely that the subjectivity is higher by using SRH, a single item on health in general, than with SF-36 questionnaire, which is a questionnaire with valid items focusing people on specific aspects of health. Therefore it may be a less subjective measure than SRH and less exposed to variability associated with individual health expectations. Moreover quality of life is a broader concept than SRH. Several dimensions of life are important, such as subjective well-being, happiness, life satisfaction or social relationships and networks [51]. The notion of resources is probably in part at the origin of this contrast between the two indicators. While perceived health depends on expectations and on comparison with peers, quality of life refers back to an analysis close to that of handicap in opposition to incapacity. Quality of life estimates in a broad way how a disease or disability influences social functioning. In this respect, the notion of financial, social and cultural resources becomes essential to deal with the health conditions. In this context, a high level of resources could limit the impact of a disease on quality of life [52].

## Conclusions

In conclusion, the relationship between subjective health and health conditions is influenced by SEP. This influence depends on the indicators used to measure socioeconomic position. Compared with people in low socioeconomic position, among socially advantaged people some health conditions seem to have a relatively greater impact on SRH, but decrease quality of life to a lesser extent. Therefore, when aiming to analyze social inequalities in health, the use of subjective health indicators could underestimate (SRH) or overestimate (HRQoL) the magnitude of health inequalities existing between socioeconomic groups. Subjective health indicators are not equivalent measures and cannot be used interchangeably. They do not present the same stability as mortality and should be used with caution for analyzing health disparities.

## Abbreviations

SRH: Self-Rated Health; HRQoL: Health Related Quality of Life; SEP: Socioeconomic Position; FL: Functional Limitations; PCS: Physical Component Summary; MCS: Mental Component Summary

## Competing interests

The authors declare that they have no competing interests.

## Authors' contributions

CD, VLC and TL conceived and designed the study. CD and MMP built the database and variables used for the analysis. CD conducted the analysis, helped by BL and VLC and wrote the first draft. MKI and GD contributed to the interpretation of the results and writing the final manuscript. All the authors approved the final version.

## Pre-publication history

The pre-publication history for this paper can be accessed here:

http://www.biomedcentral.com/1471-2458/12/19/prepub
